# The role of muscle strength on tendon adaptability in old age

**DOI:** 10.1007/s00421-018-3947-3

**Published:** 2018-08-07

**Authors:** D. Holzer, G. Epro, C. McCrum, J. Doerner, J. A. Luetkens, L. Scheef, G. M. Kukuk, H. Boecker, A. Mierau, G.-P. Brüggemann, C. N. Maganaris, K. Karamanidis

**Affiliations:** 1 School of Sport and Exercise Sciences, Liverpool John Moores University, Liverpool, UK; 2 Sport and Exercise Science Research Centre, School of Applied Sciences, London South Bank University, 103 Borough Rd, London, SE1 0AA UK; 3 Department of Nutrition and Movement Sciences, NUTRIM School of Nutrition and Translational Research in Metabolism, Maastricht University Medical Centre+, Maastricht, The Netherlands; 4 Institute of Movement and Sport Gerontology, German Sport University Cologne, Cologne, Germany; 5 Department of Radiology, University of Bonn, Bonn, Germany; 6 Department of Exercise and Sport Science, LUNEX International University of Health, Exercise and Sports, Differdange, Luxembourg; 7 Institute of Movement and Neuroscience, German Sport University Cologne, Cologne, Germany; 8 Institute of Biomechanics and Orthopaedics, German Sport University Cologne, Cologne, Germany; 9 Cologne Center for Musculoskeletal Biomechanics, Medical Faculty, University of Cologne, Cologne, Germany

**Keywords:** Aging, Maximum muscle force, Triceps surae, Tendon stiffness, Young’s modulus, Cross-sectional area

## Abstract

**Purpose:**

The purpose of the study was to determine: (1) the relationship between ankle plantarflexor muscle strength and Achilles tendon (AT) biomechanical properties in older female adults, and (2) whether muscle strength asymmetries between the individually dominant and non-dominant legs in the above subject group were accompanied by inter-limb AT size differences.

**Methods:**

The maximal generated AT force, AT stiffness, AT Young’s modulus, and AT cross-sectional area (CSA) along its length were determined for both legs in 30 women (65 ± 7 years) using dynamometry, ultrasonography, and magnetic resonance imaging.

**Results:**

No between-leg differences in triceps surae muscle strength were identified between dominant (2798 ± 566 N) and non-dominant limb (2667 ± 512 N). The AT CSA increased gradually in the proximo-distal direction, with no differences between the legs. There was a significant correlation (*P* < 0.05) of maximal AT force with AT stiffness (*r* = 0.500) and Young’s modulus (*r* = 0.414), but only a tendency with the mean AT CSA. However, region-specific analysis revealed a significant relationship between maximal AT force and the proximal part of the AT, indicating that this region is more likely to display morphological adaptations following an increase in muscle strength in older adults.

**Conclusions:**

These findings demonstrate that maximal force-generation capabilities play a more important role in the variation of AT stiffness and material properties than in tendon CSA, suggesting that exercise-induced increases in muscle strength in older adults may lead to changes in tendon stiffness foremost due to alterations in material rather than in its size.

## Introduction

Tendons transmit muscle forces to the skeleton to allow body movement and interaction with the environment. Due to their viscoelastic behavior, tendons of the lower extremity can increase muscle efficiency during terrestrial locomotion by providing more favorable conditions for the contractile elements and by storing strain energy (Ker et al. 1987; Biewener and Roberts 2000; Hof et al. 2002; Lichtwark and Wilson 2008). Indeed, associations between tendon biomechanical properties and muscle performance capabilities have often been reported throughout the human lifespan (Arampatzis et al. 2007b; Bojsen-Møller et al. 2005; Waugh et al. 2013; Quinlan et al. 2018). Previous studies (Magnusson et al. 2008; Arampatzis et al. 2010; Seynnes et al. 2015; Wiesinger et al. 2015) have convincingly shown that despite their poor vascularity, human tendons respond to increased mechanical loading by increasing their tensile stiffness. Similar to other load-bearing structures, mechanotransduction is believed to be responsible for the tendons ability to adapt (Chiquet et al. 2009).

From a biomechanical point of view, increases in tendon stiffness can be brought about by improvements in tendon’s material (increased Young’s modulus) or tendon hypertrophy (increases in its cross-sectional area; CSA). However, previous studies show somewhat conflicting results as to which of the above two adaptive mechanisms takes place in response to changes in mechanical loading. On the one hand, medium-term exercise intervention studies lasting 12–14 weeks, which were effective in improving muscle strength, have also shown to improve tendon stiffness through a concurrent increase in both Young’s modulus and tendon CSA (Arampatzis et al. 2007a; Kongsgaard et al. 2007; Seynnes et al. 2009; Bohm et al. 2014). However, some earlier cross-sectional studies examining habitual sport-induced loading demonstrate that tendons adjust their stiffness to adapt to changes in physiological loading foremost through morphological changes rather than altering their material properties (Rosager et al. 2002; Couppé et al. 2008; Seynnes et al. 2013), which is in line with animal studies (Pollock and Shadwick 1994). In contrast, other cross-sectional and interventional studies (Bayliss et al. 2016; Kubo et al. 2001; Reeves et al. 2003; Malliaras et al. 2013) show that differences in tendon stiffness seem to be entirely or largely due to altered material properties. Furthermore, it seems that at least in younger individuals, tendon material and morphological adaptations occur over different time frames, with changes in material properties taking place earlier within an exercise training programme, whereas tendon hypertrophy appears to be a longer term adaptive response (Kjaer et al. 2009; Heinemeier and Kjaer 2011; Bohm et al. 2015a; Wiesinger et al. 2015). What leads to these diverse tendon adaptations to increased mechanical loading is not yet fully understood.

Along with a deterioration in muscle structure and function (Frontera et al. 2000), several studies have reported that the aging process is associated with a gradual decline in tendon stiffness and Young’s modulus (Karamanidis and Arampatzis 2005; Onambele-Pearson et al. 2006). This can be explained by cellular, mechanical, biochemical, and pathological changes (Noyes and Grood 1976; Vogel 1991; Kjaer 2004; Komatsu et al. 2004), which may limit the adaptability of collagenous tissue to environmental mechanical stress (Tuite et al. 1997). Furthermore, in addition to changes due to the aging process per se, alterations in mechanical stress may affect the tendon in old age. Chronically diminished physical activity, which is a common feature in old age, may reduce the mechanical stimulus required to maintain muscle size, muscle strength, and tendon properties. This notion is supported by earlier in vivo bed rest studies (20–90 days chronic inactivity) demonstrating a reduction not only in muscle strength and size, but also in tendon stiffness and Young’s modulus (Kubo et al. 2000, 2004; Reeves et al. 2005). Therefore, as a consequence of the altered mechanical environment in which aged tendons often operate, smaller contractile forces are being generated and applied to the tendon, thus reducing the mechanical stimulus which is important for preserving tendon mechanical properties. However, exercise may be effective in counteracting the deterioration in muscle–tendon unit structure and function caused by the above combined effect of the aging process and inactivity, leading to increases in muscle strength, muscle size, tendon stiffness, and tendon Young’ modulus (Reeves et al. 2003; Onambele-Pearson and Pearson 2012; Grosset et al. 2014; Karamanidis et al. 2014; Epro et al. 2017). Most exercise intervention studies in older subjects have reported an increase in Young’s modulus as the sole mechanism underpinning the increase in tendon stiffness post-training (Reeves et al. 2003; Onambele-Pearson and Pearson 2012; Grosset et al. 2014). As a consequence, contrary to previous findings in younger subjects (Arampatzis et al. 2007a; Kongsgaard et al. 2007; Seynnes et al. 2009; Bohm et al. 2014) it has been suggested that tendon hypertrophy cannot be achieved through physical exercise in old age. In marked contrast with this notion, we recently showed that exercise-induced tendon hypertrophy can take place in older adults (Epro et al. 2017). However, it must be stressed that, as opposed to previous training studies in older people, we acquired a large number of scans along the tendon, thus allowing identification of regional CSA adaptations, which might have gone undetected in earlier investigations due to the limited tendon regions scanned.

In the current study, we analyzed the ankle plantarflexor muscle strength and Achilles tendon (AT) stiffness, AT CSA, and AT Young’s modulus of older women to gain insight into the diverse adaptability of older tendons to mechanical loading, as specified in the studies above (Reeves et al. 2003; Onambele-Pearson and Pearson 2012; Grosset et al. 2014; Karamanidis et al. 2014; Epro et al. 2017). Specifically, we aimed to establish if similar to young adults, there is an association between ankle plantarflexor muscle strength (i.e., maximal AT force) and AT mechanical, material, and morphological properties in a sample of older female adults. It was hypothesized that maximal AT force would be associated with AT biomechanical properties in this group of older adults, showing higher correlation coefficients with AT Young’s modulus than with AT CSA. Moreover, we hypothesized that asymmetry between legs in maximal AT force would not be accompanied by differences in AT CSA.

## Materials and methods

### Participants and experimental setup

The study was conducted with 30 older female volunteers aged between 60 and 75 years (mean ± SD: age: 65 ± 7 years; body mass: 67 ± 9 kg; body height: 166 ± 7 cm) from a large-scale knee osteoarthritis study (*N* = 38, Kellgren–Lawrence score: 2–3) from a sub-sample of our previous study (Epro et al. 2017), who agreed to have both limbs scanned using magnetic resonance imaging (MRI). Exclusion criteria were previous AT ruptures, AT pain or injury (e.g., tendinopathy) or any other musculoskeletal impairments in the lower limbs (e.g., joint pain during locomotion) within the last 2 years. Furthermore, the assessed SF-36 general health questionnaire (average scale value 75.4%) and clinical functional tests such as SLS (single leg stance; average of 40.3 s; using test duration of 45 s) and TUG (timed up and go test; average of 7.3 s) assured that the subjects were generally healthy for their age group. In addition, all participants were taking part in some form of organized physical activity (e.g., nordic walking, hiking, swimming, bike riding, aqua-jogging, moderate resistance training), 2–3 times a week on average. After being informed about the study, all subjects gave their written consent to the experimental procedures, which were approved by the human ethics committees of the German Sport University Cologne as well as the University of Bonn (according to the Declaration of Helsinki).

To examine whether tendon properties are associated with maximal force production capacity, the first analysis considered only the individually dominant leg (preferred leg for step initiation; as in Epro et al. 2017). For investigating the inter-limb differences in AT force and AT CSA, the individually dominant leg and contralateral non-dominant leg were additionally analyzed.

### Measurement of Achilles tendon cross-sectional area

**Fig. 1:**
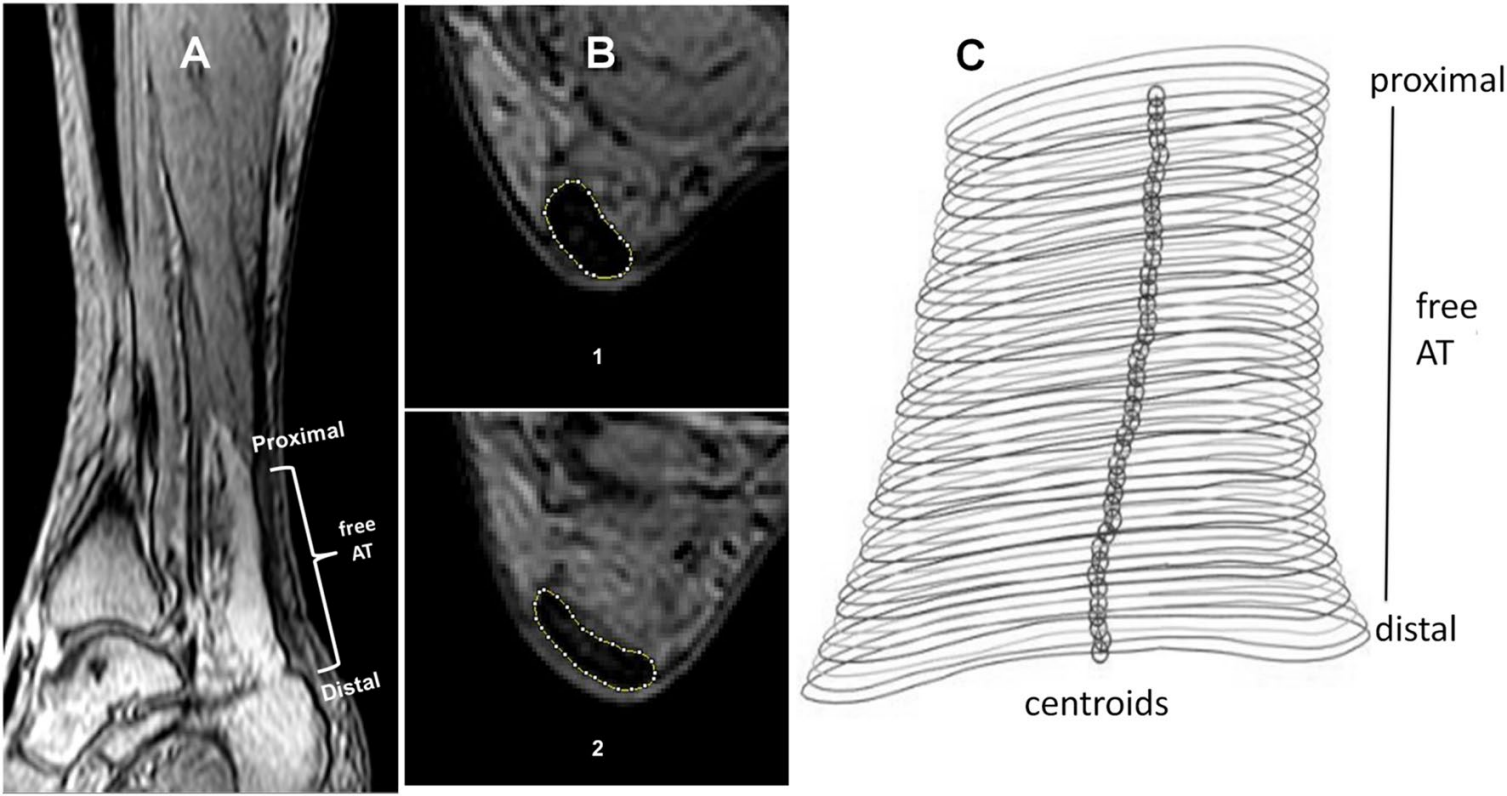
Magnetic resonance images and 3D contour plot of the free Achilles tendon (AT). Sagittal images (**a**) were used to determine the AT proximal and distal ends (M. soleus-AT junction, and AT attachment point on the calcaneum, respectively). Transversal images (**b**) between the AT proximal and distal ends were manually segmented to determine the AT cross-sectional area (CSA). Free AT length was determined as the curved path through the CSA centroids of each transversal slice (**c**)

MRI scans were obtained in a whole-body 3 T magnet (Ingenia 3T, Philips Healthcare, Best, the Netherlands) to scan and quantify the CSA of the free AT along the entire tendon length. MRI sequences were acquired in transversal and sagittal orientation using a high-resolution single-shot T1-weighted 3D gradient echo sequence (e-THRIVE). For fat suppression, an additional spectral attenuated inversion recovery (SPAIR) pulse was used. Sequence parameters were as follows: acquisition matrix = 420 × 372, acquired voxel size = 1.00 × 1.00 × 2.00 mm, reconstructed voxel size = 0.58 × 0.58 × 1.00 mm, time of repetition (TR) = 3.6 ms, time of echo (TE) = 1.7 ms, flip angle (α) = 10°, and parallel imaging factor (SENSE) = 2. During scanning the subjects lay in a supine position with both knees and hips fully extended and the ankle joints fixed at 20° plantarflexion (AT in a near-slack position; DeMonte et al. 2006). In the sagittal images recorded (Fig. 1a), the proximal and distal ends of the free AT were identified at the soleus myotendinous junction and the osteotendinous junction in the calcaneum, respectively. In every transversal-plane image (Fig. 1b) along the free AT, the tendon’s boundaries were outlined manually using the Java-based public image processing and analysis software ImageJ (National Institutes of Health, Bethesda, MD, USA). The coordinates of the AT boundaries of each image were exported and further processed using Matlab (The Mathworks, Natick, MA, USA), making it possible to create a contour plot for each AT (Fig. 1c). The length of the AT was defined as the curved path through the centroids of the single cross sections, which were determined by means of Delaunay triangulation (Bohm et al. 2016) between the two confining landmarks. The same investigator manually tracked all MRI images. Subsequently, the average CSA value of the free AT between 10 and 100% length (mean AT CSA) was determined in both legs. Furthermore, to analyze the region-specific differences in AT CSA, an average CSA was calculated for each 10% free AT length interval.

### Quantification of AT force, stiffness, and Young’s modulus

**Fig. 2:**
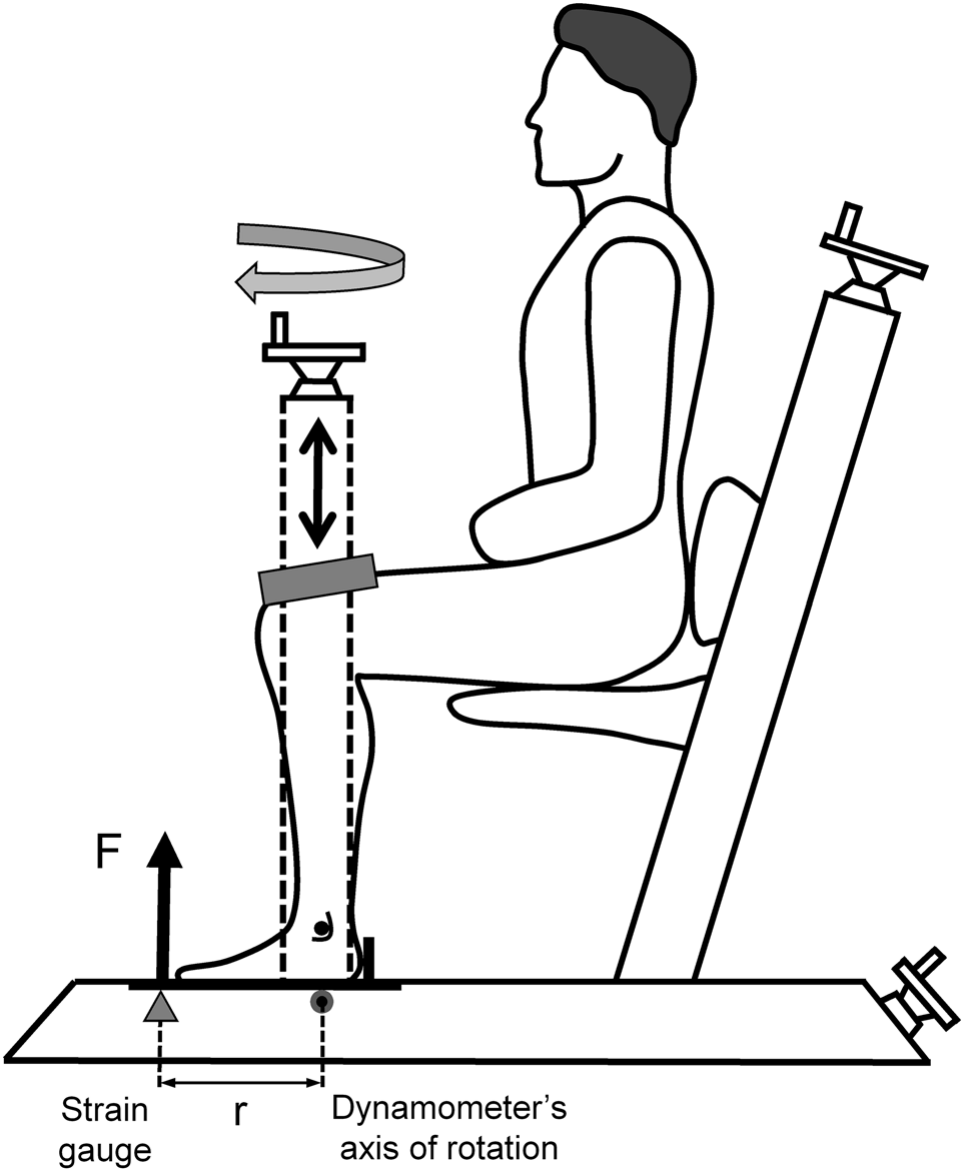
Ankle plantarflexion MVC moment was measured in a custom-made strain gauge-type dynamometer. Subjects were seated with their lower leg secured, with knee and ankle joints positioned at 90° (shank perpendicular to thigh). The foot was placed on the dynamometer so that the axis of rotation of the ankle joint was aligned with the plate’s center of rotation. Therefore, the ankle plantarflexion MVC moment was equivalent to the moment measured by the dynamometer, which is computed as the product of *F* (resultant force) and *r* (distance between the strain gauge and the dynamometer’s axis of rotation). Achilles tendon force was calculated by dividing the resultant ankle joint moment by the tendon moment arm from the dominant leg

The mechanical and material properties of the triceps surae muscle–tendon unit were assessed with the aid of synchronous ultrasonography and dynamometry. Each participant underwent a familiarization session with the measurement equipment a week prior to testing. Subjects performed three isometric ankle plantarflexion maximum voluntary contractions (MVCs) with each leg in a seated position on a custom-made strain gauge-type dynamometer (1000 Hz; please also see Epro et al. 2017), with the ankle and knee joints secured at 90° angles (thigh and foot perpendicular to the shank) and the ankle joint visually aligned with the dynamometer’s axis of rotation (Fig. 2). The resultant ankle plantarflexion moment was calculated by multiplying the force corresponding to the voltage recorded by the load-cell, by the distance between the load-cell and the dynamometer’s axis of rotation (Fig. 2). To examine the possible influence of ankle joint-dynamometer axis misalignment on the moment measured, a pilot study was conducted using a motion capturing system (120 Hz, Qualisys, Gothenburg, Sweden) in combination with a force plate (1080 Hz, 400 × 600 mm, Bertec, Columbus OH, USA) to quantify the maximal anterior displacement of the ankle joint axis during maximal isometric plantar flexion contractions. Calculated maximal anterior shift of the ankle joint axis during the contractions was on average 3.4 ± 2.1 mm, leading to a mean overestimation in the calculated joint moment by 1.7% (for more details see Ackermans et al. 2016 Supplementary Material). Before the measurement, all subjects completed a regimented warm up (2–3 submaximal plantarflexion contractions and 2–3 MVCs) with each leg. The AT force was calculated by dividing the resultant ankle joint moment by the individual tendon moment arm, which was assessed using the tendon excursion method during passive joint rotation (Maganaris et al. 2004). The AT moment arm of the dominant leg was used to calculate the AT force for both legs, as previous studies have identified no between-leg differences in its value in younger adults (Bohm et al. 2015b). For each leg, three MVC trials were performed. The highest calculated AT force out of three MVC trials (maximal AT force) was used to assess the ankle plantarflexor muscle strength for each individual leg.

Furthermore, for the dominant leg, three additional standardized MVC ramp contractions (3 s until maximum plantarflexion moment) were performed to obtain the AT force–length relationship. As the contraction duration was similar, yet the achieved joint moments differed between participants, the loading rate on the tendon was not constant within the sample. However, we recently showed that this has no effect on the tendon elongations in the upper region of the force–elongation relationship of the AT (McCrum et al. 2018a), where the stiffness measurements are typically taken in vivo. This finding is also in line with the reports of Kubo et al. (2002) and Peltonen et al. (2013). The AT elongation was measured using a securely positioned linear array ultrasound probe (29 Hz; MyLab^TM^Five, Esaote; Genoa, Italy) in the dominant limb during each ramp contraction as well as during the above mentioned passive joint rotations. The displacement of the myotendinous junction of the m. gastrocnemius medialis was manually digitized in relation to a skin marker using a video analysis software (Simi Motion 5.0, SIMI Reality Motion System GmbH, Unterschleißheim, Germany). To account for the effect of inevitable ankle joint angular rotation on the measured elongation during each contraction, a potentiometer positioned beneath the heel was used to determine changes in the ankle joint angle as described previously (Ackermans et al. 2016; Epro et al. 2017). Subsequently, the AT stiffness (mean value of the three MVC ramp contractions) was calculated as the slope of AT force and its resultant elongation relationship between 50 and 100% of maximum tendon force using linear regression. The AT Young’s modulus was determined as the slope of the AT stress–strain relationship between 50 and 100% of the maximal AT stress. The resting length of the tendon was measured as the path from the most proximal point of the tuber calcanei to the myotendinous junction of the m. gastrocnemius medialis (both determined using ultrasonography) using a flexible measuring tape along the skin surface in the same seated position at rest. The average CSA value of the free AT between 10 and 100% length (mean AT CSA) was used to calculate the AT stress.

### Statistics

Normality of distribution and homogeneity of variance in the data were confirmed using the Shapiro–Wilk and Levene’s test (*P* > 0.05). A Pearson product-moment correlation coefficient was used to examine the relationship of AT force with AT stiffness, Young’s modulus, and mean AT CSA for the dominant leg only. To examine region-specific differences in AT CSA, Pearson product-moment correlation coefficients were calculated for the relationship between maximal AT force and each 10% tendon length interval (*n* = 10; Int 10%–Int 100%). A one-way measures analysis of variance (ANOVA) was used to identify potential within-subject leg differences in maximal AT force, mean AT CSA, and in free AT length between the dominant and non-dominant legs. To consider region-specific main effects on AT CSA, a further two-way ANOVA with repeated measures was used to identify possible within-subject leg (dominant leg vs. non-dominant leg) and between tendon interval effects on AT CSA, with leg as dependent variable. Duncan’s post hoc comparison was performed when a significant main effect was detected. Furthermore, a symmetry index was determined (Robinson et al. 1987) between limbs as follows:

$${\text{Symmetry}}\;{\text{Index}}=\frac{{{X_{{\text{Dominant}}}} - {X_{{\text{Non-dominant}}}}}}{{\frac{1}{2}\left( {{X_{{\text{Dominant}}}}+{X_{{\text{Non-dominant}}}}} \right)}} \times 100\% ,$$

where *X*_Dominant_ is the parameter from the dominant limb and *X*_Non-dominant_ the corresponding parameter from the non-dominant leg. Therefore, a positive symmetry index means that the selected parameter has a higher value in the dominant than non-dominant leg, and a negative symmetry index means that the value is higher in the non-dominant leg. Potential differences between the symmetry indexes of maximal AT force and mean AT CSA as well as the individual AT CSA length intervals were analyzed using a one-way repeated measure ANOVA. Additional Pearson correlation coefficients were implemented to examine the relationship between symmetry index in maximal AT force and mean AT CSA, and between symmetry index in maximal AT force and in all AT CSA intervals. All statistical procedures were performed using Statistica (Release 10.0, StatSoft Inc., Tulsa, OK, USA) and the level of significance was set at α = 0.05. All results in the text and figures are presented as mean and standard deviation (mean ± SD).

## Results

**Fig. 3:**
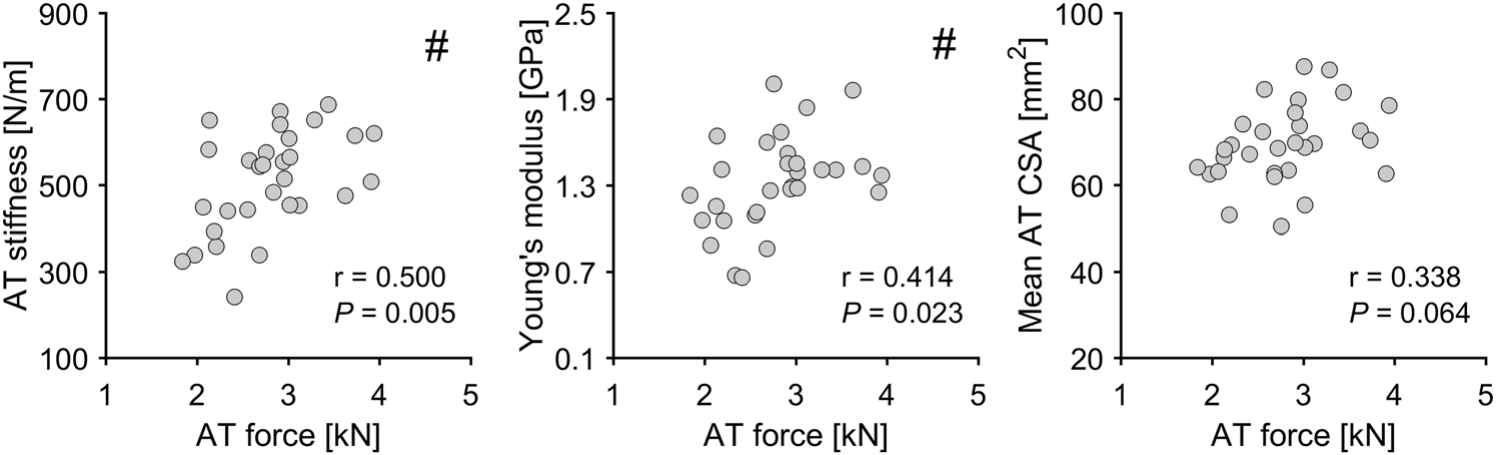
Correlations between the maximal calculated Achilles tendon (AT) force and AT stiffness, AT Young’s modulus, and mean AT cross-sectional area (CSA), respectively (*n* = 30; dominant leg). #Statistically significant correlation (*P* < 0.05)

**Fig. 4:**
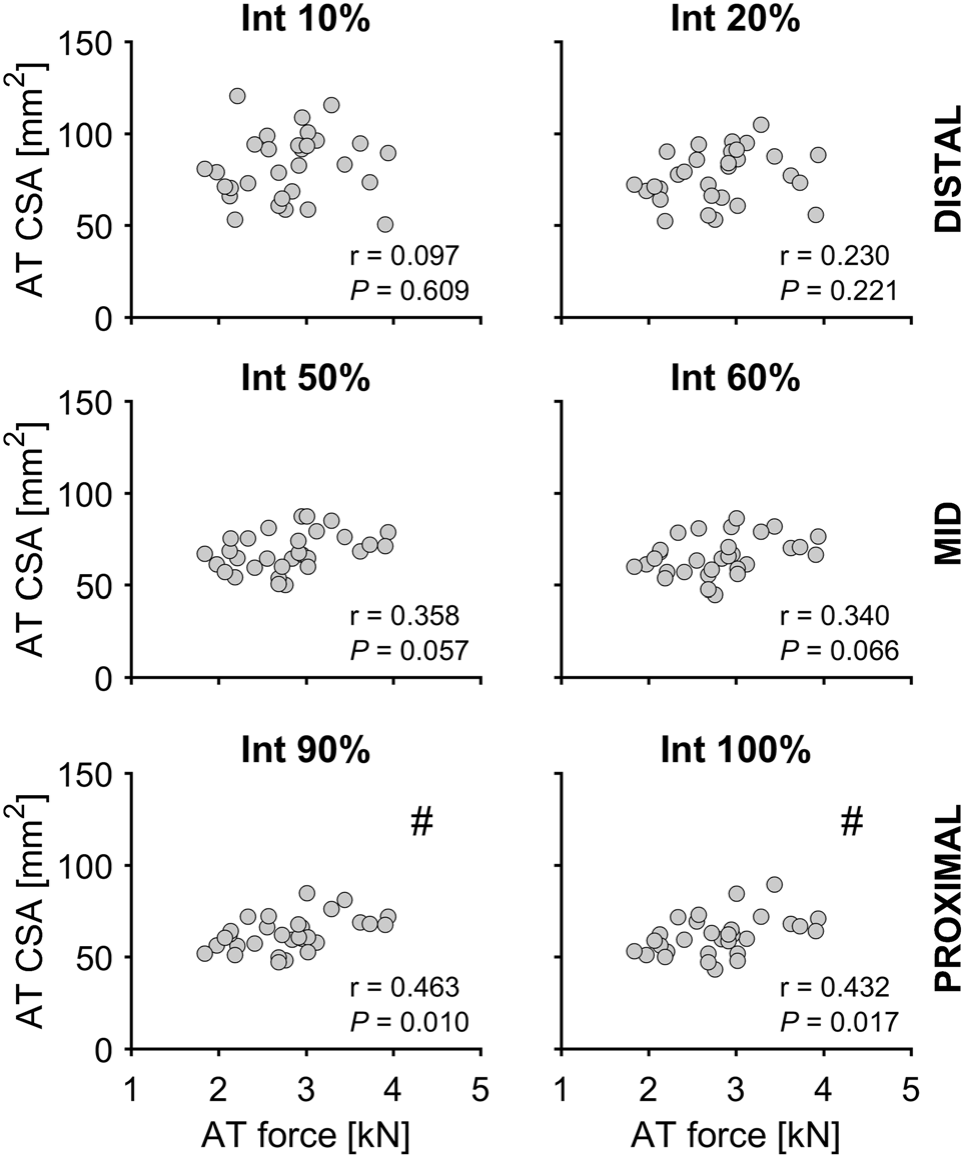
Correlations between Achilles tendon (AT) cross-sectional area (CSA) and maximal calculated AT force for the distal, mid-, and proximal portion of the AT (*n* = 30; dominant leg). #Statistically significant correlation (*P* < 0.05)

There were statistically significant correlations (*P* < 0.05; *n* = 30) between maximal AT force and AT mechanical and material properties in the dominant leg, with *r* = 0.500 and *r* = 0.414 for AT stiffness and Young’s modulus, respectively (Fig. 3). No significant correlation was found between maximal AT force and mean AT CSA (*r* = 0.338; Fig. 3). However, Pearson’s correlation coefficients between maximal AT force and AT CSA at individual tendon intervals were significant (*P* < 0.05) in the thinnest/proximal part of the tendon; Int 80%: *r* = 0.384; Int 90%: *r* = 0.463; Int 100%: *r* = 0.432 (Fig. 4).

**Fig. 5:**
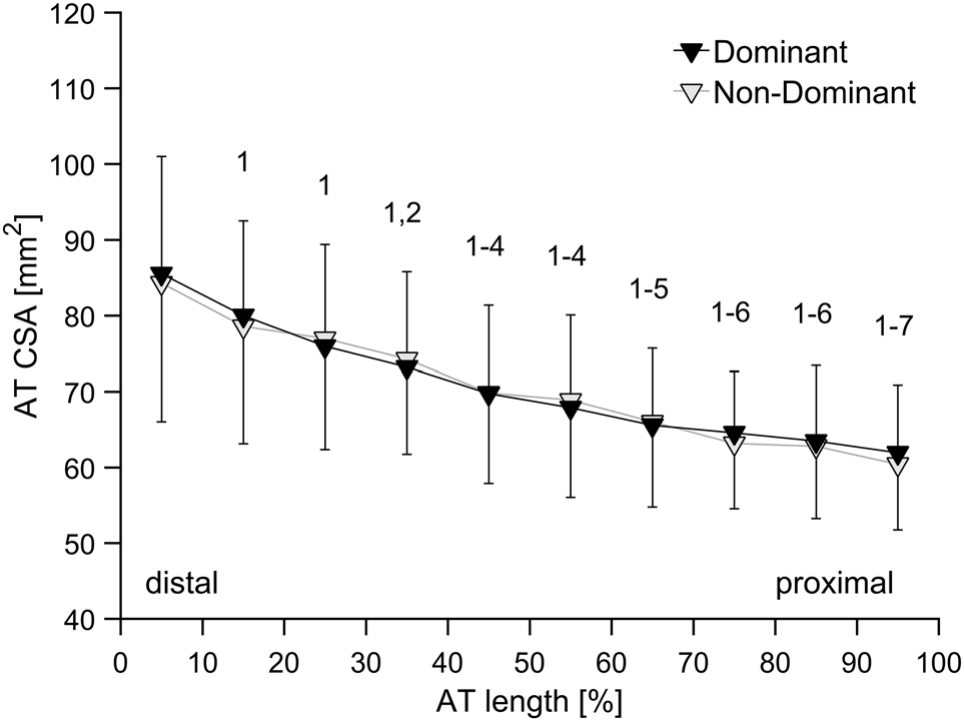
Mean and SD Achilles tendon (AT) cross-sectional area (CSA) of the examined older female adults in 10% intervals of tendon length for the dominant leg and non-dominant leg (*n* = 30). Tendon CSA is steadily increasing from the proximal to the distal end of the AT. ^1, 2, ..., 7^: statistically significant difference in CSA to Int 10%, Int 20%,..., Int 70%. (*P* < 0.05)

The within-subject leg comparison revealed no significant differences in maximal AT force between the dominant (2798 ± 566 N) and non-dominant legs (2667 ± 512 N), as well as in the length of the free AT (dominant leg: 37.0 ± 12.2 mm; non-dominant leg: 36.9 ± 11.7 mm). Regarding the AT CSA, there was a significant interval effect (*P* < 0.05), but no leg effect. Specifically, AT CSA increased from proximal toward the distal end (Fig. 5), while no significant differences in mean AT CSA (dominant leg: 71.2 ± 10.4 mm^2^; non-dominant leg: 71.4 ± 10.2 mm^2^) or analyzed AT CSA intervals were found between legs. The post hoc test showed that CSA at Int 100% was significantly (*P* < 0.05) smaller than mean CSA at Int 10–70%; Int 90% < Int 10–60%; Int 80% < Int 10–60%; Int 70% < Int 10–50%; Int 60% and Int 50% < Int 10–40%; Int 40% < Int 10–20%; and Int 30% and Int 20% < Int 10%. These differences were independent of leg (no significant interaction) (Fig. 5). The analysis of inter-limb symmetry revealed significantly higher (*P* < 0.05) symmetry index values for maximal AT force (7.2 ± 11.3%) in comparison to AT CSA (mean: − 0.3 ± 7.5%; individual length intervals: range − 3.4 to 1.4%). No significant correlations were found between the symmetry indexes of maximal AT force and mean AT CSA, or between the symmetry indexes of maximal AT force and AT CSA at the length intervals studied.

## Discussion

In the present study, we aimed to establish if there is an association between ankle plantarflexor muscle strength (maximal AT force) and AT mechanical, material and morphological properties in a sample of older female adults. Our hypothesis, that maximal AT force would be associated with AT biomechanical properties, with maximal AT force having a higher correlation with Young’s modulus than tendon CSA, was confirmed. The maximal AT force showed significant correlations with AT CSA only in the most proximal part of the tendon. Furthermore, we confirmed that the AT CSA is symmetrical between the dominant and non-dominant legs across the entire length of the tendon.

Earlier studies with younger adults have demonstrated that along with improved muscle strength in response to mechanical loading, tendons also adapt by increasing their stiffness via both alterations in material properties as well as in CSA (Arampatzis et al. 2007a; Kongsgaard et al. 2007; Seynnes et al. 2009; Bohm et al. 2014). The correlations in the current study demonstrate the importance of ankle plantarflexor muscle strength (maximal AT force) for AT stiffness and Young’s modulus also in older adults, whereby mean tendon CSA seems to be less influenced by the variation in muscle strength (*r* = 0.338; *P* = 0.064). Similar to our previous study using an unilateral analysis in older adults (Epro et al. 2017), as well as in younger adults (Arampatzis et al. 2007a, 2010; Bohm et al. 2014, 2015b), in the current study the free AT CSA showed greater CSA in the distal part of the tendon in both legs of older adults. The variation in AT CSA with length interval indicates a similar variation in tensile stresses along the tendon and as tendon adaptation is triggered by mechanical stimuli, regional adaptations should be considered. The region-specific correlations between maximal AT force and AT CSA in the current study indicate that the proximal part of the free AT may be more likely to display morphological adaptations following an increase in muscle strength in older adults. This is consistent with the application of higher tensile stress at the proximal than the distal portion of the tendon due to the smaller tendon CSA.

Taking into account cross-sectional investigations with athletes and various exercise interventions, it has been proposed (Wiesinger et al. 2015; Maganaris et al. 2017) that stiffening of tendon through modifications in its material requires certain mechanical loading characteristics (e.g., loading magnitude, frequency and/or duration), which may not necessarily occur in daily living. This rapid adaptation may continue up to a point when critical density is surpassed to facilitate tendon growth (Wiesinger et al. 2015) and further improvements in tendon stiffness would therefore be brought about by tendon hypertrophy (Maganaris et al. 2017). Due to aging-related disuse and inactivity, it may be speculated that the tendons of older people could be subjected to very high loads during some daily activities, for example during stair negotiation (Hortobágyi et al. 2003; Reeves et al. 2008; Beijersbergen et al. 2013), as the in-series muscles would be required to operate closer to their maximum strength capacities to execute the task.

**Fig. 6:**
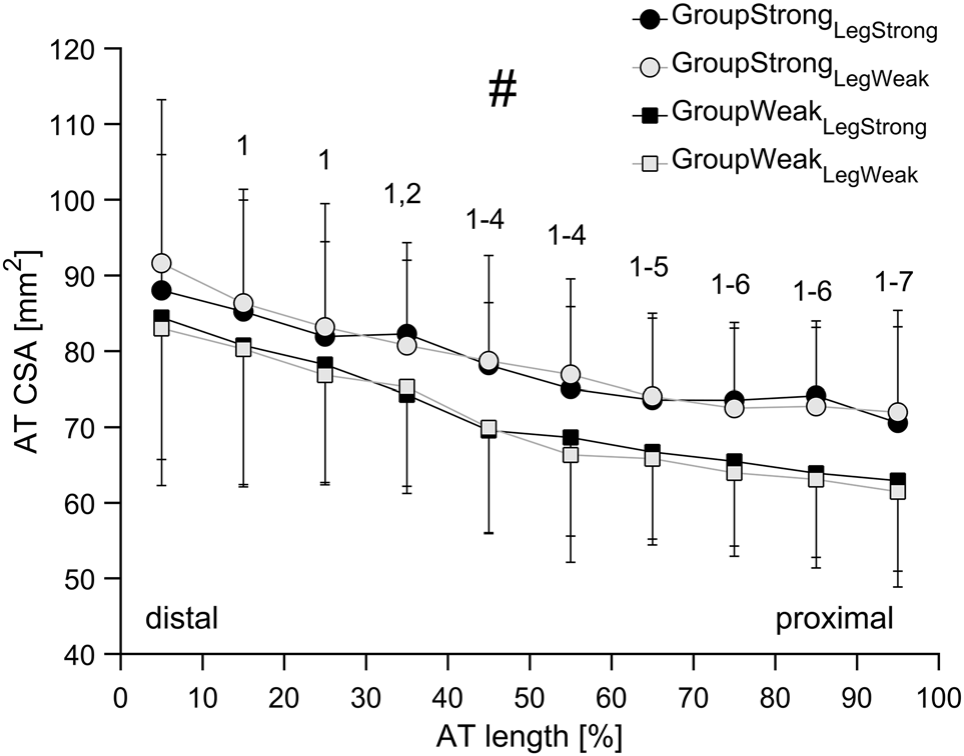
Mean Achilles tendon cross-sectional area (CSA) of the examined older female adults in 10% intervals of the tendon length for GroupStrong_LegStrong_ (*n* = 14), GroupStrong_LegWeak_ (*n* = 14), GroupWeak_LegStrong_ (*n* = 16), and GroupWeak_LegWeak_ (*n* = 16). #Statistically significant differences between the strong and weak groups (*P* < 0.05). ^1, 2, ..., 7^: statistically significant difference in CSA to Int 10%, Int 20%,..., Int 70%. (*P* < 0.05) for each leg

In the present study, no differences in AT CSA along the tendon’s length were found in the dominant compared to non-dominant leg, which may be related to a symmetry between legs in maximal AT force and a corresponding similarity in mechanical loading in daily life. This was further supported by the identified comparatively low symmetry indexes in all investigated parameters (maximal AT force, mean AT CSA as well as the individual AT CSA length intervals; range: − 3.4 to 7.2%). Previous experimental studies clearly demonstrate that habitual tendon strain caused by the transmission of muscle forces is one of the strongest indicators of risk for tensile tendon injury (Wren et al. 2003; LaCroix et al. 2013). Accordingly, it has been suggested that increasing the strength-generating capacity of a muscle would be accompanied by a modulation of the mechanical properties of the tendon (Mersmann et al. 2017). Specifically, the tendon should become stiffer when muscle strength is improving, so that the tendon remains safe and protected from a potential injury/fracture in tension caused by the increased force the muscle applies while pulling on the tendon. One possible explanation is that the tendon adapts to a change in habitual loading by increasing its stiffness through alterations in its material (Young’s modulus) rather than size (CSA). This could also explain the stronger associations between maximal AT force and AT mechanical and material properties (*r* = 0.500 for AT stiffness and *r* = 0.414 for Young’s modulus) in comparison to mean AT CSA (*r* = 0.338; *P* = 0.064) in the current study. The region-specific analysis for the AT CSA demonstrated significant relationships with maximal AT force only in the proximal part of the tendon (correlation coefficients from 0.384 to 0.463). This is in line with most exercise intervention studies with older adults, where the magnitude of post-exercise adaptations in tendon stiffness and Young’s modulus seem to be comparable to younger adults, whereas changes in tendon CSA appear to be rather limited (Reeves et al. 2003; Grosset et al. 2014; McCrum et al. 2018b). Our recent study (Epro et al. 2017), however, challenges previous results and documents that tendon hypertrophy can in fact take place in response to exercise in older people. In contrast to previous studies, we recorded a large number of scans along the tendon to detect regional tendon size adaptations that could go undetected with a limited number of scans, the typical measurement approach in previous studies. More importantly, the exercise-induced increase in muscle strength in the previous studies by Grosset et al. (2014) and Reeves et al. (2003) was smaller (9–14% muscle strength increase) than the muscle strength increase by 22–25% in Epro et al. (2017). The rather small differences in maximal AT force between legs in combination with the mere tendency toward correlation (*r* = 0.338, *P* = 0.064) between maximal AT force and average AT CSA raise the possibility that a larger improvement in muscle strength would be required for overall tendon size adaptations to occur. In the present study, the dominant leg was not always the stronger leg (in six subjects the dominant leg was the weaker leg), which means that the difference in muscle strength between dominant and non-dominant legs underestimates the muscle strength difference between the stronger and weaker legs. However, even if we account for this differentiation by considering only the 24 subjects in which the dominant leg was also the stronger leg, there is still a lack of AT CSA asymmetry between legs, despite an inter-limb difference in maximal AT force of about 12%. However, it should be noted that when dividing all subjects (*n* = 30) into a stronger and a weaker group based on a median split with average muscle strength data from both limbs, maximal AT force differences between groups were on average 36% and mean AT CSA in the stronger group was significantly larger (*P* < 0.001, ~ 9%, see Fig. 6), providing support to the notion that much higher strength differences than the present inter-limb differences would be required to bring about inter-limb differences in tendon size.

In our analysis of tendon size variation with muscle strength, we have implicitly assumed that the AT force calculated from plantarflexion moment reflects the force generated by the triceps surae muscles during MVC. This, however, is not the case, as in addition to the contracting triceps surae muscles which are joined distally to form the AT, there are six additional ankle plantarflexors that are not attached onto the AT (plantaris, tibialis posterior, flexor hallucis longus, flexor digitorum longus, peroneus brevis, and peroneus longus). Moreover, there is a “negative” moment contribution by the antagonist ankle dorsiflexors, which also co-contract during a plantarflexion MVC. However, the triceps surae muscle size occupies ~ 77% of the overall plantarflexor muscle group’s physiological CSA (Fukunaga et al. 1996) and the antagonist ankle dorsiflexors co-contract little at mid-range joint positions (Maganaris et al. 1998; Mademli et al. 2004; Arampatzis et al. 2005). Therefore, the contribution of these unaccounted factors is unlikely to explain the variation in ankle plantarflexion MVC moment (hence calculated AT force) within the sample of older women tested. In addition, we cannot exclude possible inter-limb and inter-individual differences in muscle activation level (Mademli and Arampatzis 2008; Morse et al. 2004), which might have influenced the estimation of tendon force as well as the leg-symmetry calculations. Furthermore, due to the knee joint being flexed at 90° during the measurement of maximal plantar flexion moments, it is possible that the gastrocnemius muscle was in a less favorable position to generate force in comparison to the soleus muscle. However, in our previous study (Epro et al. 2017) the same subjects showed relatively homogenous exercise-related increment in muscle thickness in soleus and gastrocnemius muscle over 14 weeks and 1.5 years by exercising exactly in the same joint configuration. In addition, we quantified the AT moment arm length in the dominant leg only, and we used this value to calculate the AT force in both legs with the reasoning that no between-leg differences have been identified in younger adults (Bohm et al. 2015b).

## Conclusions

In summary, these findings demonstrate that maximal force-generation capabilities play a more important role in the variation of AT stiffness and AT Young’s modulus than in tendon CSA, suggesting that exercise-induced increases in muscle strength in older adults may lead to changes in tendon stiffness primarily due to alterations in the tendon’s material rather than in its size. Furthermore, it seems that inter-limb asymmetries in triceps surae muscle strength are not large enough to be accompanied by morphological changes along the whole free AT in older adults.

## Abbreviations

ANOVA: Analysis of variance
AT: Achilles tendon
CSA: Cross-sectional area
MRI: Magnetic resonance imaging
MVC: Maximum voluntary contraction
SD: Standard deviation
SLS: Single leg stance
TUG: Timed up and go test
